# Safety and efficacy of an olive oil-based triple-chamber bag for parenteral nutrition: a prospective, randomized, multi-center clinical trial in China

**DOI:** 10.1186/s12937-015-0100-6

**Published:** 2015-11-14

**Authors:** Zhen-Yi Jia, Jun Yang, Yang Xia, Da-Nian Tong, Gary P. Zaloga, Huan-Long Qin, OliClinomel N4 Study Group

**Affiliations:** 1 Department of Surgery, Shanghai Sixth People’s Hospital, Affiliated to Shanghai Jiao Tong University, Shanghai, 200233 China; 2 Baxter Healthcare, Deerfield, IL 60015 USA; 3 Department of Surgery, Shanghai Sixth People’s Hospital, Affiliated to Shanghai Jiao Tong University, Shanghai, 200233 China; 4 General Surgery, Shanghai Tenth People’s Hospital (Tenth People’sHospital of Tongii University), Shanghai, 200072 China

**Keywords:** Infection, Nutritional outcomes, Olive oil-based lipid, Parenteral nutrition, Soy bean oil-based lipid, Surgery

## Abstract

**Background:**

Small studies suggest differences in efficacy and safety exist between olive oil-based (OLIVE) and soybean oil-based (SOYBEAN) parenteral nutrition regimens in hospitalized adult patients. This large, prospective, randomized (1:1), open-label, multi-center, noninferiority study compared the delivery, efficacy, and safety of OLIVE (*N* = 226) with SOYBEAN (*N* = 232) in Chinese adults (≥18 years) admitted to a surgical service for whom parenteral nutrition was required.

**Methods:**

Treatments were administered for a minimum of 5 days up to 14 days (to achieve approximately 25 kcal/kg/day, 0.9 g/kg/day amino acids, 0.8 g/kg/day lipid). Impact of treatment on anabolic/catabolic and serum inflammatory, chemistry, and hematological markers, safety, and ease of use were assessed. The primary efficacy variable was serum prealbumin level at Day 5.

**Results:**

OLIVE (*n* = 219) was not inferior to SOYBEAN (*n* = 224) based on the prealbumin least square geometric mean [LSGM] ratio [95 % CI] 1.12 [1.06, 1.19]; *P* = 0.002), improved the anabolic/catabolic status of patients enrolled in the study, and was well tolerated compared with SOYBEAN. Improved anabolic status was supported by significantly higher levels of prealbumin at Day 5, albumin at Day 5 and IGF-1 at Day 14 in the OLIVE group, while catabolism was similar between groups. C-reactive protein, intercellular adhesion molecule-1, procalcitonin, and oxidation were similar in each group, but infections were significantly lower with OLIVE (3.6 % versus 10.4 %; *P* < 0.01).

**Conclusions:**

OLIVE provided effective nutrition, was well tolerated, was associated with fewer infections, and conferred greater ease-of-use than SOYBEAN.

**Trial registration:**

NTC 01579097.

## Background

The beneficial effects of parenteral nutrition (PN) in ill patients unable to tolerate adequate enteral feeding are well established [1–3]. PN not only improves nutritional status in malnourished patients, it also reduces complications [4, 5] such as infections, need for mechanical ventilation, and muscle weakness. As a result, PN is an important therapeutic modality for treating patients with compromised intestinal function.

Animal and human studies suggest that the lipid source in PN, and more specifically the fatty-acid composition of the lipid source, may contribute to the risk of complications [6–9]. Such complications include inflammation, oxidation, immune compromise, infections, hyperglycemia, and liver dysfunction/failure. Soybean oil-based lipid emulsions are composed of long-chain triglycerides, primarily linoleic acid (ω-6 polyunsaturated fatty acids [PUFAs]). In contrast, olive oil-based lipid emulsions are composed of long-chain triglycerides, primarily oleic acid (ω-9 monounsaturated fatty-acids [MUFAs]) [10]. According to the 2012 American Society for Parenteral and Enteral Nutrition (ASPEN) Novel Nutrient Task Force, lipid sources such as safflower oil and soybean oil are considered pro-inflammatory, while olive oil is considered immune neutral [10]. Experimental reports suggest that ω-6 PUFA-rich lipid emulsions that are derived from soybean oil may exaggerate the inflammatory response associated with stress and trauma via activation of the arachidonic acid eicosanoid pathway and have direct effects on lymphocyte, macrophage, and neutrophil functions [11–14]. These ω-6 PUFA effects upon inflammation and immune cell functions may increase the rate of infections. As a result, lipid emulsions with lower linoleic acid levels (ie, olive oil predominant emulsions) have been developed. However, there are limited data to suggest that the replacement of soybean oil-based ω-6 PUFAs with olive oil-based ω-9 MUFAs improves the safety of PN [15, 16]. Therefore, one of the objectives of this study was to evaluate the incidence of infections using two different lipid emulsions that varied in the content of linoleic acid.

In this study, we utilized two different PN regimens differing in lipid emulsion type (olive oil based versus soybean oil based) and delivery system (compounded versus a ready-to-use, three-chamber bag). The primary endpoint chosen was prealbumin level (an index of anabolic and inflammatory status). Due to the lower ω-6 PUFA content (a pro-inflammatory fatty acid) and higher oleic acid content (MUFA with lower oxidation risk) of the olive-oil based lipid emulsion, we hypothesized that this emulsion would be associated with improved anabolic/catabolic status. Anabolism was assessed using prealbumin, albumin, and insulin growth factor-1 (IGF-1) levels. Catabolism was assessed using the protein breakdown end-products of urea and 3-methylhistidine. We also assessed inflammation (interleukin-6 [IL-6], C-reactive protein [CRP], intercellular adhesion molecule-1 [ICAM-1]) and oxidation (malonyldialdehyde, F2-isoprostanes) biomarkers. By measuring anabolic, catabolic, and inflammatory parameters, we were able to compare changes in anabolic parameters in patients with similar inflammatory and catabolic status. In addition to these nutritional parameters, we assessed clinical outcomes using infections (a marker of immune status), hospital length of stay, mortality, and adverse events. Safety was assessed by measuring biomarkers of organ functions (liver, renal, hematologic, and endocrine). Because a ready-to-use formulation is immediately available to clinicians (no need to compound), we also assessed preparation time.

Although several small studies have investigated the differences between soybean oil-based and olive oil-based lipid emulsions, it remains unclear whether the replacement of ω-6 PUFAs with ω-9 MUFAs results in improved efficacy and safety. To address this shortcoming, the objectives of this study were to assess the delivery, efficacy, and safety of an olive oil-based lipid PN regimen compared with a compounded soybean oil-based lipid PN regimen in Chinese adults for whom oral or enteral nutrition was not possible, insufficient, or contraindicated.

## Methods

### Study design

The study was an open-label, prospective, randomized (1:1), comparative, multi-center, active-controlled, parallel-group investigational trial (NTC 01579097) conducted in 18 centers across China. Ethics approval was obtained from the Institutional Ethics Committees and written informed consent was obtained before enrolment of any patients into the clinical trial. The study was conducted between December 29, 2011 and November 21, 2012 and was centrally administrated by Global Clinical Development & Operations (CD&O) based in Baxter Alliance Park, Belgium.

### Study population

Patients admitted to a surgical service were eligible for enrolment into the trial if all of the following criteria (and none of the exclusion criteria) were met: aged ≥18 to ≤80 years old; an inpatient that was hospitalized ≤14 days before enrolment; required PN because oral or enteral nutrition was not possible, insufficient, or contraindicated; had the capability to complete at least five days of study treatment (ie, PN); had a useable peripheral vein for intravenous (IV) delivery of PN; and was able to complete written informed consent per national regulations. Exclusion criteria were: a life expectancy of <6 days from initiation of study treatment; a known hypersensitivity to the components of the study treatments; use of prohibited medications within 30 days before enrolment; a known serious clinically significant condition such as congestive heart failure or severe renal insufficiency (renal failure that was not compensated for by hemofiltration or dialysis; patients unable to tolerate fluid load from PN); impaired hepatic function (total bilirubin >2 times the upper limit of normal; or alanine transaminase >4 times the upper limit of normal; or aspartate transaminase >4 times the upper limit of normal); a known history of human immunodeficiency virus infection; known congenital abnormalities of amino acid metabolism; known severe dyslipidemia (triglyceride level >2 times the upper limit of normal or >400 mg/dL or >4.52 mmol/L) or hyperglycemia (blood glucose >360 mg/dL); clinically significant abnormalities of plasma electrolytes; currently pregnant or lactating; prior enrolment in this clinical trial; participation in a clinical trial of any investigational drug or device concomitantly or within 30 days before enrolment in this clinical trial; or was considered unsuitable in the opinion of the Investigator. Due to clinical and resource constraints, consecutive patients were not enrolled at each center.

### Treatment protocol

Patients were randomized to either an olive oil-based lipid PN regimen using OliClinomel N4 (OLIVE; Baxter Healthcare, Deerfield, IL, USA) or a compounded soybean oil-based lipid PN regimen using Intralipid (SOYBEAN; Sino-Swed Pharmaceutical Corporation, Ltd., Wuxi City, China).

OLIVE was supplied as a ready-to-use PN product presented as a three-chamber bag (1.5 l) comprising a larger outer chamber that contains a dextrose (D-glucose) solution (final mixed concentration 80 g/l) with calcium (final mixed concentration 2 mmol/l); a middle chamber that contains a solution of 15 amino acids (final mixed concentration 22 g/L), with electrolytes including sodium (final mixed concentration 21 mmol/L), potassium (final mixed concentration 16 mmol/L), magnesium (final mixed concentration 2.2 mmol/L), and phosphate (final mixed concentration 8.5 mmol/L); and a smaller outer chamber that contains a lipid emulsion comprising 80 % olive oil and 20 % soybean oil (final mixed concentration 20 g/L).

SOYBEAN was prepared as a compounded admixture by the institutional pharmacy (1.5 l), specifically the Pharmacy Intravenous Admixture Services if available, per the investigators’ prescription. Components were manufactured by: dextrose – 20 % Glucose 500 mL (Baxter (China) Investment Co., Ltd., Shanghai, China); amino acids – amino acid 15-AA 250 mL:20 g (8 %; Hunan Kelun Pharma Co., Ltd, Yueyang City, China); and lipid – Intralipid® 10 g/dL (Sino-Swed Pharmaceutical Corporation, Ltd., Wuxi City, China). The final concentrations of dextrose, protein, and lipid were the same as for OLIVE.

Electrolytes, vitamins, and trace elements could be added to the study treatment or compounded PN based upon patient requirements. The study treatment was infused through a peripheral IV catheter via a control pump. If continued infusion via a peripheral IV was not possible, the study treatment could be infused via a peripherally-inserted central catheter or central IV line.

Study treatment was administered for a minimum of five days and up to a maximum of 14 days (either 14 days after surgery or 14 days total for patients who did not undergo surgery). Study treatment could be initiated up to three days before surgery and was administered after surgery on Day 1 at Hour 0. Preoperative PN was not mandatory and if a patient did not receive study treatment before surgery, or if the patient was not scheduled for surgery, study treatment was initiated on Day 1 at Hour 0.

From Day 0 to Day 5, patients were not to receive any food or liquid oral or enteral nutrition. The goal of treatment was to deliver 25 kcal/kg/day, 0.9 g/kg/day amino acids, and 0.8 g/kg/day lipid. The patients were allowed water and ice chips based on the clinical judgment of the Investigator.

From Day 6 through the remainder of the study treatment period, liquid oral or enteral nutrition could be added to the study treatment. The intent was to supply the total calculated daily nutritional requirement with study treatment (intravenous nutrition) plus liquid oral or enteral nutrition. Liquid oral or enteral nutrition was increased daily, as tolerated by the patient, with a concurrent reduction in study treatment, while still supplying the calculated daily nutritional requirement.

Study treatment was ceased once the patient was able to achieve ≥80 % of the calculated daily nutritional requirements by administered liquid oral or enteral nutrition, or the completion of Day 14 study treatment, whichever occurred earlier.

### Randomization

Assignment of study treatment was delegated by Baxter Healthcare Corporation to the study site pharmacist. Patient numbers were allocated using an Interactive Voice Recognition System/Interactive Web-based Recognition System according to the randomization code contained within the randomization list.

This was an open-label study. Treatment assignment was not known (ie, blinded) by the data management, biostatistical, and personnel at the central laboratory. In order to prepare the study treatment, the designated pharmacist was aware of the study treatment (unblinded). The study treatment was unblinded to the Investigator as it was standard practice that the physician and/or nurse carefully inspect the PN admixture to ensure that the integrity of the admixture was maintained throughout the length of the infusion.

### Outcome measures

The primary efficacy variable was the serum prealbumin level at Day 5. Secondary efficacy outcome(s) included preparation time of study treatment, time to achieve tolerability of oral nutrition, length of hospitalization, lipids and lipid upper derivatives (arachidonic acid, eicosapentaenoic acid [EPA], linoleic acid, oleic acid), markers of infection and inflammation (cortisol, CRP, procalcitonin, serum ICAM-1, IL-6), markers of oxidative stress (malondialdehyde, F2-isoprostane), surrogate markers of nutrition (albumin, IGF-1), urine markers of metabolism (6-h urinary urea, 3-methylhistidine), and injection site rating by the Investigator. Safety outcomes included total adverse events (AEs), liver function, and infections. Infections were based upon the clinical judgment of the investigators and utilized cultures when possible. Antibiotics were administered for all infections. Lung infections were defined as: the new onset of fever and/or leukocytosis with or without alterations in mentation, purulent sputum or organisms on smear or culture, and a new progressive or persistent infiltrate on chest X-ray consistent with pneumonia (with no other obvious cause). Scrotal infection was diagnosed by the presence of fever and/or leukocytosis, new evidence of infection in the scrotum (pain, tenderness, swelling, heat, redness), and no other recognized cause for the abnormality. Non-specific infection was diagnosed in a patient with new fever, leukocytosis, tachycardia, tachypnea and no other obvious cause for the abnormalities, and a response to antibiotic treatment. These outcomes were approved by the China State Food and Drug Administration as being sufficient to register the OLIVE product in China.

### Statistical analysis

A sample size of approximately 200 patients (98 per study treatment) would provide 90 % power to claim noninferiority between groups for prealbumin at Day 5. The primary comparison was that OLIVE was not inferior to the SOYBEAN in maintaining or increasing serum prealbumin levels. In the sample size calculation, it was assumed that the true ratio was 1, the coefficient of variance (CV) was 0.5, and the non-inferiority margin was −20 %. These assumptions resulted in a sample size of 98 patients per study treatment. It was decided to enroll at least 200 patients per treatment group with an evaluable primary endpoint to generate additional clinical data for this study. The sample size was calculated by PASS version 2011. Approximately 500 subjects were randomized to achieve 400 subjects who had efficacy assessments on Day 5, assuming up to 20 % of randomized subjects would drop out of the study before the Day 5 assessments.

The primary comparison was that OLIVE is not inferior to SOYBEAN in maintaining or increasing serum prealbumin levels. Noninferiority was claimed if the anti-log of the lower bound of the 95 % confidence interval (CI) of the treatment difference was at least 0.80.

Analyses were performed on the intention-to-treat population (ITT), defined as all patients randomized. The primary efficacy analyses were based on the modified intention-to-treat (mITT) population, defined as all ITT patients who received study treatment and provided some efficacy data. The per protocol (PP) population was defined as the subset of ITT patients who had no major protocol violations and had Day 5 efficacy data. All safety analyses were conducted on the safety population, defined as all patients who received any amount of study treatment.

Unless otherwise stated, all statistical analyses were performed using a two-sided hypothesis test at the 5 % level of significance. No adjustment for Type I error rate was required for the analysis of the primary endpoint because the study had only one primary endpoint with one comparison. Due to the large sample size there was no need to test the model assumptions (ie, normality test).

The primary efficacy variable was the serum prealbumin level at Day 5. The log transformed primary efficacy variable was analyzed via an analysis of covariance (ANCOVA) model with treatment and study site as the main effects and baseline serum prealbumin as the covariate. The least square (LS) mean ± standard deviation (SD) estimates of the treatment effects for OLIVE and the control, as well as the two-sided 95 % CI of the geometric mean ratio of test/control was derived.

Preparation time of study treatment (Days 1 to 5) and length of hospitalization were summarized by treatment group. Differences between treatment groups were analyzed using Kruskal-Wallis test. Time to achieve tolerability of oral nutrition was summarized using the Kaplan-Meier method and compared using a log-rank test. Other secondary efficacy variables were analyzed the same way as the primary variable with change from baseline and ANCOVA model on log-scale with baseline as covariate and treatment and study site as main effects. Injection site rating by the Investigator was analyzed using the Cochran-Mantel-Haenszel test with modified ridit scores.

For the safety analyses, statistical comparisons were performed between the two treatment groups. Continuous data were compared using an ANOVA and categorical data were compared using a Chi-square test/Fisher's exact test. AE relationship and severity were compared using Cochran-Mantel-Haenszel test with modified ridit scores. AEs were coded according to the Medical Dictionary for Regulatory Activities (MedDRA).

All statistical analyses were completed using SAS version 9.2 (SAS Institute Inc, Cary, North Carolina USA).

## Results

### Patient disposition

**Figure 1:**
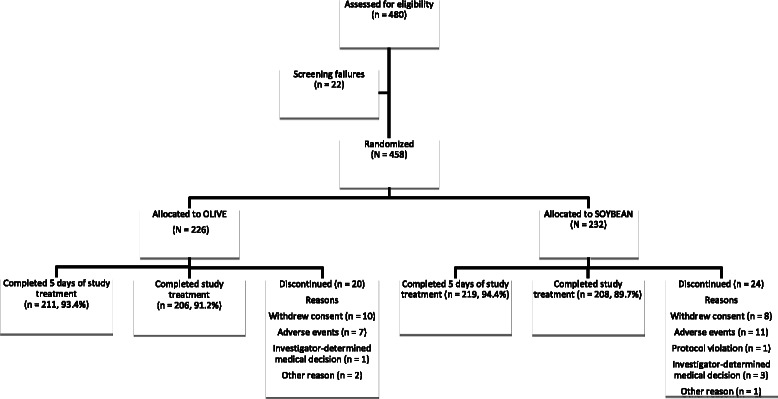
Patient disposition flowchart

A total of 480 patients consented (Fig. 1) and 458 patients were enrolled and randomized into the study. All randomized patients were included in the ITT population, 226 (100.0 %) patients in the OLIVE group and 232 (100.0 %) patients in the SOYBEAN group. The safety population comprised 222 (98.2 %) patients in the OLIVE group and 231 (99.6 %) patients in the SOYBEAN group. The mITT population comprised 219 (96.9 %) patients in the OLIVE group and 224 (96.6 %) patients in the SOYBEAN group. The PP population comprised 183 (81.4 %) patients in the OLIVE group and 190 (81.9 %) in the SOYBEAN group. Similar percentages of patients discontinued from the study in the OLIVE group (8.8 %) and the SOYBEAN group (10.3 %). The main reasons for discontinuation in both groups were patient withdrew consent and AEs (Fig. 1).

### Demographic and baseline clinical characteristics

The demographic and baseline clinical characteristics of patients in the OLIVE and the SOYBEAN groups (ITT population) were not different (Table 1). Most patients were male (61 %, 279/458), identified as Chinese Han (95 %, 436/458), and had a mean age of 56 years. Most patients underwent surgery and experienced high complexity surgery (62 %, 283/458) with a mean duration of 3 h.

**Table 1:** Patient Demographics and Baseline Characteristics of the Intention to Treat Population

Variable	OLIVE (*n* = 226)	SOYBEAN (*n* = 232)	*P* Value
Sex, n (%)			0.482
Male	134 (59.3)	145 (62.5)	
Female	92 (40.7)	87 (37.5)	
Race, n (%)			0.673
Chinese Han	216 (95.6)	220 (94.8)	
Chinese other minority	8 (3.5)	11 (4.7)	
Other	2 (0.9)	1 (0.4)	
Age, years, mean ± SD	55.8 ± 13.1	56.3 ± 11.7	0.656
BMI, kg/m^2^, mean ± SD	21.7 ± 3.9^a^	21.8 ± 3.9^b^	0.667
Underwent surgery, n (%)	195 (86.3)	202 (87.1)	0.805
Complexity of surgery^c^, n (%)			0.859
Medium complexity	49 (21.7)	48 (20.7)	
High complexity	140 (61.9)	143 (61.6)	
Missing	37 (16.4)	41 (17.7)	
Duration of surgery, hours, mean ± SD	2.9 ± 1.3	3.0 ± 1.4	0.645

*Abbreviations*: *BMI* body mass index, *SD* standard deviation

^a^*n* = 217

^b^*n* = 226

^c^Complexity of surgery was determined by the investigator based on patient and surgical characteristics

### Nutritional intakes

Treatment exposure and doses administered were similar between the treatment groups. The mean ± SD durations of study treatment exposure were 8.4 ± 3.7 days in the OLIVE group and 8.2 ± 3.7 days in the SOYBEAN group. The mean ± SD doses administered were 16761.0 ± 7086.8 mL in the OLIVE group and 16877.5 ± 7453.0 mL in the SOYBEAN group. There were no statistically significant differences in the mean ± SD flow rate, total volume prescribed, total volume administered, volume ratio (prescribed/administered volume), total duration prescribed, total duration administered, or duration ratio (prescribed/administered duration) between the treatment groups (data not shown). There was no statistically significant difference between the time to achieve adequate enteral intake (following 5 days of PN) for the OLIVE group and the SOYBEAN group (median time: OLIVE 2.0 days versus SOYBEAN 2.0 days; log rank *P* = 0.786).

### Anabolic/catabolic endpoints

**Figure 2:**
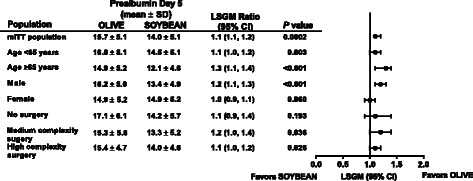
OLIVE efficacy in the modified intention-to-treat population and prespecified subgroups. Mean ± SD prealbumin levels on Day 5. LSGM ratio and 95 % CI for the comparison of OLIVE versus SOYBEAN. The LSGM ratio is the antilog of (log (GM) ± 1.96SE). The *P* value for group difference was adjusted for baseline values and study sites. *Abbreviations*: *CI* confidence interval, *LSGM* least square geometric means, *mITT* modified intention-to-treat, *PN* parenteral nutrition, *SD* standard deviation, *SE* standard error

OLIVE was noninferior to SOYBEAN in maintaining or increasing serum prealbumin levels at Day 5 in the mITT population (LSGM ratio and [95 % CI] 1.12 [1.06, 1.19]; *P* = 0.0002) and the PP population (LSGM and [95 % CI] 1.12 [1.05, 1.19], *P* = 0.0006) (Fig. 2). This observation was consistent in the subgroup analyses of age, gender, no surgery, surgery of medium complexity, and surgery of high complexity (Fig. 2).

Serum prealbumin levels and serum albumin levels were significantly higher in the OLIVE group compared with the SOYBEAN group at Day 5 (Table 2). Prealbumin and albumin increased in the OLIVE group and decreased in the SOYBEAN group. No significant difference in serum IGF-I levels were observed between the treatment groups at Day 5; however, at end of therapy (EOT)/Day 14, serum IGF-I levels were significantly higher in the OLIVE group compared with the SOYBEAN group. No significant between-group differences were observed in the 6-h urinary urea nitrogen and 6-h urinary excretion of 3-methylhistidine (Table 2).

**Table 2:** Effects of OLIVE and SOYBEAN on Markers of Nutrition in Patients Receiving Parenteral Nutrition

Nutrition Marker		OLIVE		SOYBEAN		OLIVE vs SOYBEAN	
	Visit	*N*	Mean ± SD	*N*	Mean ± SD	LSGM Ratio (95 % CI)	*P*-value
Albumin (g/L)	Baseline	214	33.11 ± 5.75	220	33.23 ± 5.84		.
	Day 5	211	34.02 ± 5.32	216	32.93 ± 5.15	1.04 (1.01, 1.07)	0.0139
	EOT/Day 14	82	35.37 ± 5.86	78	35.55 ± 5.3	1 (0.96, 1.05)	0.9393
Prealbumin (mg/dL)	Baseline	213	15.08 ± 4.64	219	15.15 ± 5.01		.
	Day 5	217	15.66 ± 5.12	218	13.95 ± 5.05	1.12 (1.06, 1.19)	0.0002
	EOT/Day 14	210	17.24 ± 6.82	219	15.15 ± 6.37	1.16 (1.08, 1.24)	0.0001
Prealbumin (PP) (mg/dL)	Baseline	182	14.82 ± 4.58	188	14.97 ± 5.02		.
	Day 5	184	15.7 ± 5.01	190	14.19 ± 5.06	1.12 (1.05, 1.19)	0.0006
	EOT/Day 14	184	17.38 ± 6.96	193	15.37 ± 6.43	1.16 (1.07, 1.25)	0.0002
IGF-I (ug/L)	Baseline	214	107.31 ± 49.61	220	110.9 ± 55.34		.
	Day 5	211	125.81 ± 64.35	218	121.15 ± 61.11	1.04 (0.96, 1.13)	0.3437
	EOT/Day 14	82	142.9 ± 70.16	78	125.27 ± 65.22	1.16 (1, 1.35)	0.045
6-h urea nitrogen, urine (mmol/L)	Baseline	211	124.02 ± 92.1	218	129.39 ± 78.47		.
	Day 5	211	141.95 ± 100.85	216	151.82 ± 98.15	0.91 (0.8, 1.03)	0.1271
	EOT/Day 14	81	150.4 ± 100.19	76	144 ± 101.81	1 (0.82, 1.23)	0.9797
6-h 3-Methylthistidine, urine (ug/mL)	Baseline	211	1.32 ± 2.61	217	1.37 ± 1.56		.
	Day 5	212	1.44 ± 4.29	216	1.11 ± 0.98	1.02 (0.9, 1.14)	0.7791
	EOT/Day 14	81	1.98 ± 2.18	77	1.74 ± 4.4	1.1 (0.87, 1.38)	0.4224

*Abbreviations*: *CI* confidence interval, *EOT* end of therapy, *IGF-I* insulin-like growth factor-I, *LSGM* least square geometric mean, *PP* per protocol, *SD* standard deviation

### Lipid endpoints

Significant increases in serum oleic acid levels were observed for both treatment groups; however, the increases in the OLIVE group were greater than those observed in the SOYBEAN group (Table 3). No statistically significant differences in serum levels of linoleic acid, arachidonic acid, and EPA were observed between treatment groups at any timepoint (Table 3).

**Table 3:** Effects of OLIVE and SOYBEAN on Serum Free Fatty Acid Levels in Patients Receiving Parenteral Nutrition

Free Fatty Acid		OLIVE		SOYBEAN		OLIVE vs SOYBEAN	
	Visit	*N*	Mean ± SD	*N*	Mean ± SD	LSGM Ratio (95 % CI)	*P*-value
Arachidonic Acid (ng/mL)	Baseline	214	2582.36 ± 1628.43	219	2435.61 ± 1691.17		.
	Day 5	211	1647.86 ± 1169.39	217	1657.64 ± 1109.07	0.96 (0.83, 1.1)	0.5511
	EOT/Day 14	82	1907.33 ± 1503.86	78	2061.49 ± 1368.59	0.82 (0.64, 1.06)	0.127
Eicosapentaenoic Acid (ng/mL)	Baseline	214	6238.86 ± 10452.35	219	5162.16 ± 3904.17		.
	Day 5	211	5492.24 ± 4259.41	217	5490.93 ± 3916.9	1.05 (0.91, 1.21)	0.5148
	EOT/Day 14	82	5604.48 ± 5433.61	78	4451.74 ± 3218.58	1.04 (0.75, 1.43)	0.8314
Linoleic Acid (ng/mL)	Baseline	214	114371.89 ± 81968.03	219	106834.77 ± 74014.98		.
	Day 5	211	122618.72 ± 74778.06	217	138023.51 ± 84529.99	0.89 (0.77, 1.04)	0.1543
	EOT/Day 14	82	120846.77 ± 101009.54	78	122106.26 ± 73206.47	0.76 (0.53, 1.09)	0.1369
Oleic Acid (ng/mL)	Baseline	214	70758.12 ± 85210.57	219	63562.45 ± 44727.06		.
	Day 5	211	85697.52 ± 52610.19	217	71773.72 ± 45452.38	1.22 (1.09, 1.37)	0.0006
	EOT/Day 14	82	81704.84 ± 58732.76	78	63696.58 ± 45050	1.11 (0.81, 1.51)	0.517

*Abbreviations*: *CI* confidence interval, *EOT* end of therapy, *LSGM* least square geometric mean, *SD* standard deviation

### Inflammation, oxidation, and infections

There was a very small but significant difference at Day 5 in the serum levels of IL-6 observed between the OLIVE and the SOYBEAN groups (Table 4). In both groups, IL-6 levels decreased. No significant differences in the serum levels of cortisol, procalcitonin, CRP, or ICAM-1 were observed between treatment groups. No significant differences in the serum levels of malondialdehyde or F2-isoprostane were observed at Day 5 or EOT/Day 14 (Table 4).

**Table 4:** Effects of OLIVE and SOYBEAN on Markers of Inflammation and Infections in Patients Receiving Parenteral Nutrition

Marker		OLIVE		SOYBEAN		OLIVE vs SOYBEAN	
	Visit	*N*	Mean ± SD	*N*	Mean ± SD	LSGM Ratio (95 % CI)	*P*-value
Cortisol (nmol/L)	Baseline	214	492.98 ± 284.67	219	499.61 ± 256.24		.
	Day 5	212	371.97 ± 145.68	218	362.98 ± 130.29	1.02 (0.95, 1.1)	0.5677
	EOT/Day 14	82	382.37 ± 193.88	78	395.31 ± 155.08	0.9 (0.77, 1.04)	0.1617
C-Reactive protein (mg/L)	Baseline	214	54.56 ± 54.46	220	56 ± 51.13		.
	Day 5	211	42.33 ± 42.9	216	47.17 ± 42.98	0.95 (0.81, 1.12)	0.5503
	EOT/Day 14	82	20.95 ± 34.44	78	21.92 ± 31.23	0.9 (0.62, 1.31)	0.5785
Procalcitonin (ng/mL)	Baseline	214	0.91 ± 1.84	219	1.19 ± 2.67		.
	Day 5	212	0.36 ± 0.97	218	0.44 ± 1.29	0.98 (0.86, 1.11)	0.717
	EOT/Day 14	82	0.15 ± 0.2	78	0.28 ± 1.1	0.88 (0.71, 1.11)	0.283
sICAM-1 (ug/L)	Baseline	214	264.37 ± 127	220	260.97 ± 128.62		.
	Day 5	209	321.56 ± 158.86	217	304.41 ± 154.53	1.02 (0.94, 1.1)	0.6462
	EOT/Day 14	81	337.84 ± 152.58	78	352.73 ± 197.27	0.96 (0.85, 1.08)	0.4683
Interleukin-6 (ng/L)	Baseline	214	143.03 ± 271.46	220	191.72 ± 705.37		.
	Day 5	211	19.63 ± 57.58	218	20.11 ± 44.19	0.82 (0.69, 0.96)	0.0173
	EOT/Day 14	ND	ND	ND	ND	ND	ND
Malondialdehyde (nmol/L)	Baseline	214	12.46 ± 25.2	219	11.4 ± 21.93		.
	Day 5	211	13.57 ± 27.65	217	12.49 ± 23.13	0.97 (0.87, 1.08)	0.5865
	EOT or Day 14	82	12.65 ± 27.81	78	10.77 ± 21.68	0.86 (0.71, 1.04)	0.115
F2-Isoprostane (ng/mL)	Baseline	214	0.13 ± 0.17	219	0.11 ± 0.14		.
	Day 5	211	0.19 ± 0.33	217	0.14 ± 0.2	1.13 (0.85, 1.5)	0.4006
	EOT/Day 14	82	0.16 ± 0.21	78	0.18 ± 0.29	0.9 (0.58, 1.4)	0.6502

*Abbreviations*: *CI* confidence interval, *EOT* end of therapy, *LSGM* least square geometric mean, *ND* not detected, *SD* standard deviation, *sICAM* serum intercellular adhesion molecule-1

Significantly more patients in the SOYBEAN group (10.4 % [24/231]) than the OLIVE group (3.6 % [8/222]) experienced an infection or infestation (Table 5). Overall, the frequency of infections in the study was low (7.1 % [32/453]). The most common infections were lung infections, which were higher in the SOYBEAN group. The second most common infection was incision/wound infections. Bloodstream infections were not reported to occur in the study.

**Table 5:** Treatment Emergent Infections

Infection, n (%)	OLIVE (*n* = 222)	SOYBEAN (*n* = 231)
Total infections	8	26*
Total patients infected	8 (3.6 %)	24* (10.4 %)
Lung infections	2	13*
Incision/wound site infections	5	3
Urinary tract infections	1	2
Abdominal/gastrointestinal infections	0	2
Scrotal infections	0	1
Nonspecified infections (systemic infection, site not identified)	0	5

*P <0.01

### Preparation time

**Figure 3:**
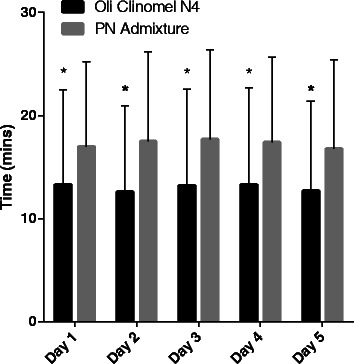
Preparation time of study treatment (Day 1 through Day 5).*P <0.05 by Kruskal-Wallis. Error bars indicate standard deviations

The preparation time for study treatment was significantly less for OLIVE compared with the SOYBEAN on all days assessed (*P* <0.001 for all values [Fig. 3]).

### Hospital time and mortality

There were no significant differences between the OLIVE group and the SOYBEAN group for the length of hospitalization from admission to discharge or for the length of hospitalization from randomization to discharge (Table 6). Five patients died during the study; one patient in the OLIVE group (cardiopulmonary failure) and four patients in the SOYBEAN group (severe pharyngeal hemorrhage, severe abdominal infection, severe hypovolemic shock and severe acute renal failure).

**Table 6:** Effects of OLIVE and SOYBEAN on Hospital Time in Patients Receiving Parenteral Nutrition

Length of Hospitalization	OLIVE			SOYBEAN			OLIVE vs SOYBEAN
	*N*	Mean ± SD	Median	*N*	Mean ± SD	Median	*P*-value
From admission to discharge, days	219	16.92 ± 4.99	16	224	18.1 ± 8.65	16	0.7823
From randomization to discharge, days	219	10.84 ± 4.27	10	224	12.04 ± 7.82	10	0.4854

*Abbreviations*: *SD* standard deviation

### Safety endpoints

#### Safety and tolerability

Overall, the safety and tolerability profile of OLIVE was similar to SOYBEAN. The most common treatment-emergent AEs (TEAEs) occurring in greater than 5 % of patients in both groups were pyrexia and infusion site swelling (Table 7). Discontinuations from the study were low, with seven patients in the OLIVE group and nine patients in the SOYBEAN group discontinuing from the study due to AEs.

**Table 7:** Study Drug-Related Adverse Events in the Safety Population

System Organ Class Preferred Term	OLIVE		SOYBEAN	
	Frequency^a^	n (%)^b^	Frequency^a^	n (%)^b^
General disorders and administration site conditions	164	85 (38.3)	157	89 (38.5)
Swelling	60	19 (8.6)	50	17 (7.4)
Pyrexia	38	33 (14.9)	40	35 (15.2)
Infusion site swelling	20	16 (7.2)	26	24 (10.4)
Gastrointestinal disorders	65	43 (19.4)	72	50 (21.6)
Nausea	22	17 (7.7)	19	17 (7.4)
Vascular disorders	47	33 (14.9)	65	41 (17.7)
Phlebitis	24	12 (5.4)	31	12 (5.2)
Peripheral vascular disorder	14	13 (5.9)	23	22 (9.5)
Metabolism and nutrition disorders	35	28 (12.6)	60	42 (18.2)
Hypokalemia	3	3 (1.4)	18	18 (7.8)
Investigations	41	27 (12.2)	38	26 (11.3)
Gamma-glutamyltransferase increased	11	11 (5.0)	7	7 (3.0)
Injury, poisoning and procedural complications	44	27 (12.2)	30	25 (10.8)
Procedural pain	21	12 (5.4)	9	7 (3.0)
Incision site pain	16	12 (5.4)	12	10 (4.3)
Respiratory, thoracic and mediastinal disorders	31	23 (10.4)	26	20 (8.7)
Productive cough	14	14 (6.3)	11	11 (4.8)
Cough	11	11 (5.0)	11	11 (4.8)
Infections and infestations	8	8 (3.6)	26	24 (10.4)
Hepatobiliary disorder	13	13 (5.9)	7	7 (3.0)

^a^Number of events

^b^Number and percentage of patients with at least one event

There were no differences between groups in the severity of TEAEs (*P* = 0.073) or in the relationship of TEAEs to study treatment (*P* = 0.393) as considered by the Investigators. Most TEAEs were considered to be mild in severity in both the OLIVE group (77.6 % [125/222] patients) and the SOYBEAN group (69.8 % [118/231] patients). Severe TEAEs were reported in 2/222 (1.2 %) patients in the OLIVE group and 10/231 (5.9 %) patients in the SOYBEAN group. The severe TEAEs reported by the two patients in the OLIVE group were cardiopulmonary failure and acute myocardial infarction. The severe TEAEs reported by the ten patients in the SOYBEAN group were epistaxis, pharyngeal hemorrhage, urinary tract infection, abdominal infection, hypovolemic shock, lung infection, pyrexia, hypokalemia, hypernatremia, ureteric fistula, renal failure acute, vomiting, and intestinal fistula.

Eight patients in the OLIVE group and 14 patients in the SOYBEAN group experienced serious AEs (SAEs). Overall, each SAE was reported by ≤1.1 % of patients. All reported SAEs were considered by the Investigator to be not associated or unlikely to be related to the study treatment. Most SAEs were considered by the Investigator to be moderate or severe and recovered/resolved with no sequelae by the end of the study.

#### Systemic safety

Alanine aminotransferase and aspartate aminotransferase were higher in the SOYBEAN group at baseline (Table 8). There were no statistically significant differences between groups at Day 5 or EOT/Day 14 and mean values were within normal limits or only slightly above the normal range. However, when analyzed for change from baseline, alanine aminotransferase was significantly different between groups at Day 5 and EOT/Day 14, whereas aspartate aminotransferase was not significantly different between groups. Alkaline phosphatase was similar between groups at baseline. Levels were significantly different between groups at Day 5 but not at EOT/Day 14. Most values were within normal ranges. Gamma-glutamyltransferase was within normal limits for most patients and similar between groups at baseline. When analyzed by change from baseline, gamma-glutamyltransferease values were higher in the OLIVE group than in the SOYBEAN group at Day 5, but not at EOT/Day 14. Total bilirubin was similar between groups at all time points. Blood urea nitrogen was similar between groups at baseline. When analyzed by change from baseline, blood urea nitrogen values were higher in the OLIVE group at Day 5 but not at EOT/Day 14. Most values remained within normal limits throughout the study. Creatinine remained normal in both groups throughout the study; there were no differences between groups.

**Table 8:** Absolute Serum Chemistry Measures in the Modified Intention to Treat Population

Serum Chemistry Measure		OLIVE		SOYBEAN		OLIVE vs SOYBEAN	
	Visit	*N*	Mean ± SD	*N*	Mean ± SD	LS Mean ± SE (95 % CI)	*P*-value
Alanine aminotransferase, U/L	Baseline	212	41.4 ± 87.15	216	73.3 ± 197.14		
	Day 5	79	31.0 ± 27.86	85	29.2 ± 55.41	21.3 ± 6.89 (7.7, 34.9)	0.002
	EOT/Day 14	201	48.8 ± 61.23	196	38.5 ± 38.70	42.5 ± 15.24 (12.5, 72.5)	0.006
Alkaline phosphatase, U/L	Baseline	210	72.5 ± 46.87	212	71.7 ± 35.65		
	Day 5	79	87.7 ± 58.27	85	74.7 ± 40.41	18.8 ± 5.65 (7.7, 30.0)	0.001
	EOT/Day 14	201	123 ± 96.24	196	106.9 ± 70.89	11.6 ± 7.72 (−3.6, 26.8)	0.133
Aspartate aminotransferase, U/L	Baseline	210	52.8 ± 117.38	212	72.3 ± 191.38		
	Day 5	79	31.9 ± 19.27	85	22.6 ± 15.82	22.4 ± 8.87 (4.9, 40.0)	0.12
	EOT/Day 14	201	35.8 ± 39.42	196	30.6 ± 35.13	25.5 ± 17.25 (−8.5, 59.4)	0.141
γ-Glutamyltransferase, U/L	Baseline	210	39.2 ± 74.63	212	42.7 ± 70.32		
	Day 5	79	100.5 ± 136.62	85	68.9 ± 71.69	34.9 ± 11.96 (11.3, 58.5)	0.004
	EOT/Day 14	201	139.2 ± 165.89	196	118.1 ± 140.65	12.4 ± 13.45 (−14.1, 38.8)	0.358
Total bilirubin, μmol/L	Baseline	212	18.00 ± 15.98	216	16.12 ± 10.69		
	Day 5	79	13.21 ± 9.76	85	11.86 ± 9.56	−0.39 ± 1.50 (−3.35, 2.56)	0.793
	EOT/Day 14	201	12.86 ± 9.34	196	13.87 ± 18.94	−2.97 ± 1.42 (−5.76, −0.19)	0.36
Blood urea nitrogen, mmol/L	Baseline	212	3.63 ± 2.07	216	3.88 ± 1.91		
	Day 5	85	4.21 ± 2.51	92	4.35 ± 2.71	0.51 ± 0.24 (0.03, 0.99)	0.039
	EOT/Day 14	200	5.23 ± 2.47	195	4.93 ± 2.27	0.38 ± 0.21 (−0.03, 0.79)	0.072
Creatinine, μmol/L	Baseline	212	67.8 ± 23.6	218	67.4 ± 21.1		
	Day 7	110	62.0 ± 15.8	112	60.2 ± 17.4	−2.3 ± 2.7 (–7.5, 3.0)	0.40
	EOT/Day 14	200	62.6 ± 16.5	195	62.2 ± 16.7	−0.7 ± 1.7 (-4.1, 2.7)	0.69
Chloride, mmol/L	Baseline	212	104.4 ± 4.33	216	103.7 ± 4.39		
	Day 5	85	101.4 ± 3.55	93	102.4 ± 4.53	−1.6 ± 0.73 (−3.1, −0.2)	0.027
	EOT/Day 14	200	101.0 ± 3.55	194	101.8 ± 5.03	−1.3 ± 0.59 (−2.5, −0.1)	0.030
Phosphorus, mmol/L	Baseline	155	1.07 ± 0.26	162	1.15 ± 0.79		
	Day 5	83	1.21 ± 0.47	89	0.98 ± 0.45	0.25 ± 0.09 (0.09, 0.42)	0.003
	EOT/Day 14	183	1.26 ± 0.21	176	1.20 ± 0.32	0.17 ± 0.08 (0.00, 0.34)	0.044
Potassium, mmol/L	Baseline	212	4.58 ± 9.07	216	3.98 ± 0.47		
	Day 5	85	4.35 ± 0.57	93	3.95 ± 0.53	0.43 ± 0.10 (0.23, 0.63)	<0.001
	EOT/Day 14	201	4.97 ± 9.16	194	4.06 ± 0.53	0.28 ± 0.06 (0.15, 0.40)	<0.001
Total cholesterol, mmol/L	Baseline	206	3.52 ± 0.95	210	3.60 ± 0.89		
	Day 5	77	3.48 ± 0.96	84	7.60 ± 38.15	−4.35 (−13.47, 4.77)	0.348
	EOT/Day 14	196	3.76 ± 0.90	188	3.6 ± 0.94	0.20 (0.02, 0.39)	0.029
Triglycerides, mmol/L	Baseline	206	0.89 ± 0.53	211	0.91 ± 0.58		
	Day 5	77	1.89 ± 0.95	85	1.51 ± 0.68	0.41 (0.19, 0.63)	<0.001
	EOT/Day 14	200	1.72 ± 0.88	193	1.50 ± 0.67	0.25 (0.09, 0.40)	0.002

*Abbreviations*: *CI* confidence interval, *EOT* end of treatment, *LS* least squares, *SD* standard deviation, *SE* standard error

For the change from baseline in serum electrolytes, statistically significant differences in chloride (Day 5 and EOT/Day 14), phosphorus (Day 5 and EOT/Day 14), and potassium (Day 5 and EOT/Day 14) were observed (Table 8). Bicarbonate, chloride, phosphorus, potassium, and sodium levels were within normal limits in both treatment groups at Day 5 and EOT/Day 14.

Cholesterol increased more in the OLIVE group compared to the SOYBEAN group, but values remained within normal limits in most patients (Table 8). Triglyceride levels increased in both groups, reflecting the triglyceride content of the lipid emulsions. Values were slightly, but significantly higher in the OLIVE group compared to the SOYBEAN group. No patient withdrew from the study due to lipid abnormalities (ie, elevated cholesterol or triglycerides) and none of the PN infusions required adjustment for lipid levels.

No statistically significant differences (all *P* >0.05) between the OLIVE group and SOYBEAN group were observed for serum glucose or insulin use (Table 9). In addition, there were no significant differences between groups for insulin levels in the blood or hematological measures (basophils, neutrophils, eosinophils, lymphocytes, monocytes, platelets, hemoglobin, and hematocrit). Absolute counts for basophils, eosinophils, lymphocytes, monocytes, and neutrophils remained within normal ranges in both treatment groups (data not shown).

**Table 9:** Serum Glucose Concentrations (mmol/L) and Insulin Use^a^

	*n*	OLIVE Mean ± SD	*n*	SOYBEAN Mean ± SD	*P*-value (change from baseline between groups)
Baseline	212	7.1 ± 2.3	216	7.2 ± 2.3	
Day 3	207	8.6 ± 3.1	218	8.4 ± 3.5	0.74
Day 7	110	6.7 ± 2.6	112	7.0 ± 2.7	0.13
EOT or Day 14	201	6.3 ± 2.2	193	6.9 ± 3.0	0.09
Patients requiring insulin	41 (18.5 %)	–	48 (20.8 %)	–	

^a^Values are not significantly different at any time point; *Abbreviations*: *EOT* end of treatment, *SD* standard deviation

## Discussion

This is the largest prospective, randomized, open-label, controlled, multi-center study to date that compares two lipid emulsions used as part of complete PN; an olive oil-based lipid emulsion PN regimen and a soybean oil-based lipid emulsion PN regimen. Overall, OLIVE was not inferior to SOYBEAN for prealbumin levels, improved the anabolic/catabolic status of patients enrolled in the study, and was well tolerated compared with SOYBEAN. The improvement in anabolic status for the OLIVE group was indicated by higher levels of the protein endpoints of prealbumin, albumin, and IGF-1. The lack of difference in urinary excretion of urea and 3-methylhistidine between the groups suggests that catabolism was similar in each group. CRP, ICAM-1, procalcitonin, and oxidation were similar in each group, but infections were significantly lower with OLIVE. Oleic acid levels increased with OLIVE; but linoleic acid, arachidonic acid, and EPA were similar between groups. OLIVE was associated with shorter preparation times compared with the compounded SOYBEAN admixture.

Prealbumin was chosen as the primary endpoint for the study following discussion with a number of experts. Serum prealbumin levels are determined by a variety of factors, including synthesis, degradation, and tissue distribution. Synthesis is dependent upon supply of amino acids to the liver and activation of prealbumin synthetic pathways. During metabolic stress, as occurs with illness and surgery, activation of inflammatory pathways (especially IL-6) increase prealbumin degradation, limit it’s synthesis, and may increase tissue distribution by leakage into tissues (increased vascular permeability) [17]. Lipid emulsions and other components of PN have been shown to modulate endothelial permeability, inflammation, anabolism, catabolism, and immune cell functions [18]. As a result, prealbumin was thought to be a composite indicator of amino acid supply, protein synthetic capacity, catabolism, and inflammation. All of these endpoints are important targets for nutrition support and were assessed in this study. Anabolism was assessed with prealbumin, albumin, and IGF-1 levels; catabolism was assessed with nitrogen excretion and 3-methylhistidine excretion. In addition, we also assessed other endpoints that included infections, hospital stay, morbidities (AEs), mortality, organ function (renal, hepatic, hematologic), and metabolic status (oxidation, fatty acids, cholesterol, triglycerides, glucose, insulin, electrolytes).

OLIVE was associated with significant increases in prealbumin, albumin, and IGF-I compared with SOYBEAN. As no difference between groups for catabolic markers were noted, these results would suggest that the effects of OLIVE are likely to be mediated by anabolism. IGF-I is particularly sensitive to protein intake and responds rapidly to protein energy status [19]. This is the first study to compare the effects of an olive oil-based PN regimen with a soybean oil-based PN regimen on serum IGF-I levels. While numerous studies have reported the positive nutritional effects of OLIVE on one or perhaps two nutritional markers [8, 20–22], this is the first study to examine both anabolic and catabolic marker profiles and one of the first studies to demonstrate that OLIVE is effective in increasing anabolism in critically ill patients. The exact mechanism for the differences in anabolism are unclear, but may relate to the high ω-6 PUFA levels in the SOYBEAN versus OLIVE emulsion that may exacerbate the inflammatory response to surgery and illness.

The physiologic response to injury is an acute phase reaction (APR) mediated by a sudden increase in cortisol and the production of proteins such as CRP and cytokines (IL-1, IL-6, tumor necrosis factor-α [TNFα]). APR proteins have direct effects on catabolism and inflammatory pathways and, therefore, play a role in nutritional status, particularly in critically ill patients where malnutrition and systemic inflammation coexist [19]. Both OLIVE and SOYBEAN were associated with decreases in cortisol, CRP, and IL-6 levels. The effects of OLIVE and SOYBEAN on APR proteins were consistent with a prior smaller study that showed both lipid formulations were associated with decreases in CRP and TNFα [21]. Prior studies have also shown that OLIVE significantly lowers IL-6 levels compared with a medium chain triglyceride/long chain triglyceride (MCT/LCT) lipid emulsion, but no between-group differences in IL-6 were noted in two studies that compared OLIVE with SOYBEAN [14, 21, 23]. A limited number of acute phase proteins (ie, CRP, IL-6, procalcitonin, ICAM-1, cortisol) were assessed in this study and no differences between levels were observed. Oxidative markers (ie, MDA, F2-isoprostane) were also measured and no differences were found. It is possible that more frequent sampling, measurement of tissue levels rather than blood levels, or measurement of other markers (ie, leukocyte functions) may have shown differences between lipid groups.

SOYBEAN lipid emulsion contains high levels (approximately 54 %) of the ω-6 PUFA, linoleic acid, while OLIVE contains much lower levels (approximately 18 %). It has been hypothesized that the high levels of linoleic acid in SOYBEAN exacerbate inflammation [15, 16, 18, 24, 25] in patients undergoing acute illness and tissue injury (such as major surgery). However, we were unable to detect such exacerbation in this study using conventional inflammatory markers such as cortisol, IL-6, ICAM-1, CRP, and procalcitonin. It is possible that other inflammatory markers (such as leukotrienes) may have been higher in the SOYBEAN group than in the OLIVE group.

Small studies directly comparing olive oil-based lipid emulsions with soybean oil-based lipid emulsions have provided conflicting results, with some studies showing significant reductions in inflammatory and oxidative stress markers [23], while other studies have reported no differences in inflammatory or oxidative stress markers between olive oil-based PN and soybean oil-based PN admixtures [14, 21]. Furukawa and colleagues [12] investigated the impact that the degree of stress associated with surgery had on IL-6 production in patients receiving lipid-free or soybean oil-based PN admixtures. The results of their study showed that soybean oil-based PN significantly increased IL-6 levels in patients experiencing severe stress, but not in patients experiencing moderate stress. Several reasons may account for the differences between prior studies and this study including sample size, differences in the patient populations between the studies, or differences in the level of stress the patients were experiencing.

Measurement of lipids and lipid upper derivatives revealed changes in the levels of fatty acid moieties that were consistent with the lipid source contained in the two PN regimens. Not surprisingly, linoleic acid levels increased in both treatment groups, albeit it to a lesser extent in the OLIVE group. Despite the trend for increased linoleic acid in the SOYBEAN group, arachidonic acid levels decreased similarly in both groups. Thus, supply of larger amounts of linoleic acid with SOYBEAN had no effect upon circulating arachidonic acid levels. The decrease in arachidonic acid levels in both groups suggests that it was being consumed as substrate for the APR. It is important to note that tissue levels of these fatty acids, not measured in this study, may better reflect cellular effects of the fatty acids. Similarly, the significantly greater increase in oleic acid observed in the OLIVE group compared with the SOYBEAN group is consistent with the olive oil contained in OLIVE. Cholesterol and triglycerides are components of the lipid emulsions used in both groups. Cholesterol increased to a greater extent in the OLIVE group; however, differences in levels were small and unlikely to have clinical significance. Cholesterol levels remained within normal limits (ie, <5.2 mmol/L) in most patients. Triglyceride levels increased in both study groups, reflecting the infusion of triglycerides in the lipid emulsions. Levels increased slightly but significantly more in the OLIVE group. Most levels remained below the upper recommended triglyceride level for parenteral nutrition (ie, 3.5–4.5 mmol/L). The greater increase in triglycerides in the OLIVE group likely reflects the slightly slower clearance of olive oil-based triglycerides compared to soybean triglycerides.

One of the most clinically relevant findings of this study was the decreased incidence of infections in the OLIVE group compared to the SOYBEAN group. There were many similarities between the two groups including rate and complexity of surgery, duration of stay in hospital, duration of PN, and glucose levels. Therefore, these factors do not explain the difference in infection rate.

Studies comparing compounded PN with multi-chamber PN have shown that compounded PN is associated with a higher rate of bloodstream infections [26]. However, in this study we did not see a difference in blood stream infections between groups, as most infections were of pulmonary origin followed by incision site infections and urinary tract infections. Small studies have also reported that the use of soybean oil-based lipid emulsions is associated with an increased risk of infections [15]. This effect is believed to be a result of the proinflammatory and immunosuppressive effects of the high linoleic acid content of soybean oil-based emulsions [15, 16, 27]. However, it should be noted that the overall rate of infections in this study was low, as is typically seen in elective surgery populations. Historically, infection rates in patients receiving total PN have been much higher. Prior studies have suggested that lipid emulsions that are high in oleic acid may have less impact on the immune system compared with soybean oil-based lipid emulsions [15, 17, 28, 29]. In contrast, a recent study comparing SOYBEAN with OLIVE reported that there were no differences in immune cell functions in 100 intensive care unit (ICU) patients [21]. Thus, the causes or mechanisms responsible for the different incidences of infection between groups remains unclear.

Manzanares et al. [30] performed a systematic review of randomized clinical studies comparing lipid emulsions with low versus high linoleic acid (n-6 polyunsaturated fatty acid) levels in critically ill patients. However, patients with elective surgery were excluded from the analyses. Thus, the results of this systematic review may not be applicable to elective surgery patients (studied in the present trial). Overall, the lower linoleic acid formulations in the studies demonstrated trends toward lower mortality (relative risk [RR] 0.83, 95 % CI 0.62, 1.11, *P* = 0.2), duration of mechanical ventilation (−2.57 days, 95 % CI −5.51, 0.37, *P* = 0.09), and ICU length of stay (−2.31 days, 95 % CI −5.28, 0.66, *P* = 0.13). There was no difference between formulations for total infections (RR 1.13, 95%CI 0.87, 1.46, *P* = 0.35). Four of the 12 studies in the review used an olive oil based lipid emulsion and compared it to MCT/LCT (2 trials) or soybean emulsion (2 trials). In this subset of studies, there was no difference between groups for mortality or ICU length of stay. Total infections tended to be higher in the olive group (RR 1.23, 95 % CI 0.92, 1.63, *P* = 0.16) whereas ventilation time was significantly lower in the olive group (−6.47, 95 % CI −11.4, −1.5, *P* = 0.01).

The preparation time of PN solutions was significantly less for OLIVE than for the SOYBEAN PN admixture. One of the goals of this study was to assess current methods of PN delivery in Chinese ICUs. Historically, lipid emulsions have been administered separately from the PN solution because of the storage requirements for the emulsion. With the advent of multi-chamber PN systems it is now possible to provide a simultaneous and continuous infusion of all nutrients using a single pump and IV line [31]. Evidence suggests that the use of these systems reduces the risk of microbial contamination due to reduced handling, reduces the risk of bloodstream infections, decreases the risk of error, decreases nursing time, and decreases risk of physical instability of the PN mixture [31–33]. Health economic investigations also suggest that multi-chamber PN systems can reduce costs associated with the manpower required to prepare compounded PN admixtures [34, 35]. When compounding PN admixtures, special attention needs to be paid to aseptic technique, the order of mixing, the presence of residual air in the bag, the type of bag material, and the storage conditions in order to prevent failure or contamination of the admixture. These factors all affect the amount of preparation time needed to prepare these solutions [36, 37]. Further, preparation of compounded admixtures requires specially trained staff and/or specialized automated equipment, whereas use of pre-prepared multi-chamber bag systems only requires proper inspection of the solutions before initiation of the infusion [38].

No differences in the clinical outcomes of length of hospitalization, mortality, or time to achieve tolerability of oral nutrition were observed between the treatment groups. However, other clinically relevant outcomes such as quality of life, functional status, or rehospitalization rates were not assessed in this study. Several small studies have reported that olive oil-based parenteral nutrition is associated with reductions in the duration of mechanical ventilation [22, 39] and length of stay in the ICU [22]. In contrast, other larger studies have reported no difference in the length of hospital stay or ICU length of stay between olive oil-based and soybean oil-based admixtures [21, 26]. However, it is likely that the differences observed between the current study and prior studies relate to the small sample sizes or to the specific patient populations enrolled in the studies.

OLIVE was well tolerated and no significant differences in the frequency or severity of TEAEs were observed between the treatment groups. In addition, the frequency of TEAEs leading to discontinuation of study drug was low. The incidence of hepatobiliary AEs was low and not different between groups. The liver enzymes, alanine aminotransferase and aspartate aminotransferase, were different between groups at baseline but were not significantly different at Day 5 or EOT/Day14. However, when analyzed by change from baseline, values decreased more in the SOYBEAN group, reflecting the higher baseline levels. The cause for the higher baseline levels most likely reflects differences in the underlying disease of the patients. The normalization of these liver enzymes following surgery suggests that they were related to the underlying disease. Alkaline phosphatase and gamma-glutamyltransferase values were similar at baseline. Levels were significantly higher in the OLIVE group at Day 5 but not at EOT/Day 14. Day 5 values reflected only a portion of the patients. Mean values for alkaline phosphatase were within normal limits or minimally elevated. Values for gamma-glutamyltransferase were elevated in both groups at EOT/Day 14. The increase in both enzymes suggests that both PN regimens were associated with mild cholestasis. Cholestasis has been associated with long-term use of PN; however, it is unclear whether the changes reflect the use of PN or reflect changes following abdominal surgery. Importantly, total bilirubin was similar between groups and most patients had values within normal limits. The clinical relevance of these findings is unclear. Perhaps a longer duration of PN would have revealed additional information. Whether these early changes are important in the context of long-term PN cannot be determined from this study; however, short-term PN with OLIVE or SOYBEAN did not appear to negatively impact liver function.

Clinical chemistry measures remained within normal limits in both treatment groups for the duration of the study. Small statistically significant differences were noted between groups and once again, the clinical relevance remains unclear. Of note, potassium and phosphorus levels were better maintained within normal ranges in the OLIVE group compared to the SOYBEAN group. The better maintenance of phosphorus levels may have resulted from the use of organic phosphorus in OLIVE compared with inorganic phosphorus in SOYBEAN. Organic phosphorus has a longer circulation time than inorganic phosphorus since it is not directly filtered in the urine, allowing more time for entry of phosphorus into cells. Overall, the results of the study suggest that OLIVE has a safety profile that makes it suitable for use in ill patients requiring PN.

The internal and external validity of this study are strengthened by the prospective, randomized, controlled, and multi-center study design. Although the study was open label, which may have contributed to bias in the reporting of treatment effects, it was not feasible to conduct a blinded study because it is standard practice that the physician and/or nurse carefully inspect the PN formulations to ensure that the integrity of the admixture (technically an emulsion) is maintained throughout the length of the infusion. In addition, the study is one of the largest (*N* = 458) to compare the efficacy and safety of two PN regimens in well-matched study populations and there was consistency in the findings across a variety of endpoints. The maximum duration of PN administration was 14 days, which is typical for elective surgery patients who usually receive PN for 7 to 10 days following surgery, as enteral feeding is gradually increased. However, the relatively short duration of follow-up (a maximum of 14 days) may be considered a limitation. Despite this, it is possible that a longer duration of PN administration may have resulted in additional differences between the treatment groups.

In conclusion, OLIVE improved the anabolic/catabolic status of hospitalized adults compared to SOYBEAN and was well tolerated. OLIVE was non-inferior to SOYBEAN for prealbumin and significantly increased prealbumin, albumin, and IGF-I serum levels to a greater extent than SOYBEAN. Inflammation and oxidation were similar in each group, but infections were significantly lower with OLIVE. The results of this study suggest that an olive oil-based PN regimen improves nutritional outcomes, lowers infections, is well tolerated, and confers greater ease-of-use compared with a compounded soybean oil-based PN regimen.

## Abbreviations

AE: Adverse event
CI: Confidence interval
CRP: C-reactive protein
CV: Coefficient of variance
EOT: End of therapy
EPA: Eicosapentaenoic acid
ICAM-1: Intercellular adhesion molecule-1
ICU: Intensive care unit
IGF-1: Insulin-like growth factor-1
IL-6: Interleukin-6
ITT: Intention-to-treat population
IV: Intravenous
LS: Least square
LSGM: Least square geometric mean
mITT: Modified intention-to-treat population
MUFA: ω-9 monounsaturated fatty-acids
PN: Parenteral nutrition
PP: Per protocol
PUFA: ω-6 polyunsaturated fatty acids
SAE: Serious adverse event
SD: Standard deviation
TEAE: Treatment-emergent adverse event
TNFα: Tumor necrosis factor-α
